# An Overview of the ADReSS-M Signal Processing Grand Challenge on Multilingual Alzheimer’s Dementia Recognition Through Spontaneous Speech

**DOI:** 10.1109/ojsp.2024.3378595

**Published:** 2024-03-18

**Authors:** SATURNINO LUZ, FASIH HAIDER, DAVIDA FROMM, IOULIETTA LAZAROU, IOANNIS KOMPATSIARIS, BRIAN MACWHINNEY

**Affiliations:** 1 Usher Institute, Edinburgh Medical School, The University of Edinburgh, EH16 4UX Edinburgh, U.K.; 2 School of Engineering, The University of Edinburgh, EH9 3JW Edinburgh, U.K.; 3 Department of Psychology, Carnegie Mellon University, Pittsburgh 15213, PA USA; 4 Information Technologies Institute, CERTH, Thessaloniki, Thermi-Thessaloniki 57001, Greece

**Keywords:** Biomedical signal processing, medical conditions, Alzheimer’s disease, human disease biomarkers, speech processing, natural language processing, multilingual Alzheimer’s dementia detection

## Abstract

The ADReSS-M Signal Processing Grand Challenge was held at the 2023 IEEE International Conference on Acoustics, Speech and Signal Processing, ICASSP 2023. The challenge targeted difficult automatic prediction problems of great societal and medical relevance, namely, the detection of Alzheimer’s Dementia (AD) and the estimation of cognitive test scoress. Participants were invited to create models for the assessment of cognitive function based on spontaneous speech data. Most of these models employed signal processing and machine learning methods. The ADReSS-M challenge was designed to assess the extent to which predictive models built based on speech in one language generalise to another language. The language data compiled and made available for ADReSS-M comprised English, for model training, and Greek, for model testing and validation. To the best of our knowledge no previous shared research task investigated acoustic features of the speech signal or linguistic characteristics in the context of multilingual AD detection. This paper describes the context of the ADReSS-M challenge, its data sets, its predictive tasks, the evaluation methodology we employed, our baseline models and results, and the top five submissions. The paper concludes with a summary discussion of the ADReSS-M results, and our critical assessment of the future outlook in this field.

## I. INTRODUCTION

There has been a great increase in interest in signal processing and machine learning methods for the detection of Alzheimer’s and other forms of dementia through analysis of speech [1], [2]. While approaches to assessing cognitive function, including dementia and mild cognitive impairment detection, have increasingly employed deep learning methods [3], other efforts focus on identifying speech features that indicate cognitive changes [4].

Machine learning models of disease detection and prognostic assessment have been proposed but often lack standardisation and common benchmarks against which the different approaches and models could be compared [2]. This situation has improved somewhat in recent years with the increasing availability of speech and language data sets for dementia research [5], [6], [7], and the advent of machine learning shared tasks (“grand challenges”) in Alzheimer’s detection through spontaneous speech [8], [9]. While many of the approaches proposed in the context of those challenges produced high accuracy results based on the analysis of spontaneous speech [10], [11], the data employed were limited to American English data. Moreover, even where classification and regression methods were based on acoustic, as opposed to language-dependent features, it was unclear whether such acoustic analysis approaches would generalise across languages [12]. In order to investigate this question, we organised the ADReSS-M Challenge at ICASSP 2023, which targeted dementia detection across two languages [13].

Alzheimer’s Dementia (AD) is a category of neurodegenerative syndromes that entails a long-term and usually gradual decrease of cognitive functioning. To diagnose and assess disease progression as well as cognitive decline, biomarkers are often employed. A biomarker (or biological marker) is, in the U.S. Food and Drug Administration (FDA) definition, “a defined characteristic that is measured as an indicator of normal biological processes, pathogenic processes or responses to an exposure or intervention” [14]. Unfortunately, most existing biomarkers for AD are either costly (neuroimaging methods such as positron emission tomography, PET, or magnetic resonance imaging, MRI) or invasive (such as analytes extracted from cerebrospinal fluid, which involve a lumbar puncture procedure). Alternative assessment methods, such as standardised cognitive tests, often suffer from ceiling effects [15], and are subject to daily fluctuations that affect cognition and executive function.

As cost-effective and accurate biomarkers of neurodegeneration have been sought in the field of dementia research, speech-based “digital biomarkers” have emerged as a promising possibility. Speech seems particularly well suited for this task, as speech and language convey much information about one’s cognitive function and can be collected in natural settings and over time thus overcoming the daily fluctuations caused by fatigue, low mood, short-term illnesses and text anxiety, which tend to affect the reliability of cognitive test performance. However, as noted, the general applicability of speech-based digital biomarkers depends on whether they can be deployed in different linguistic contexts. This question has been under-researched in this emerging field. The “ADReSS-M: Multilingual Alzheimer’s Dementia Recognition through Spontaneous Speech” challenge sought to enable the investigation of this issue by defining prediction tasks whereby participants trained their models on English speech data and assessed those models’ performance on spoken Greek data. One should note, however, that in contrast to traditional biomarkers, which have been treated as individual features in risk models [16], the speech biomarkers investigated in this challenge are better seen as composite biomarkers, consisting of the combination of multiple metrics into a single multivariate model [17]. The models submitted to the challenge investigated acoustic and linguistic features of the speech signal whose predictive power were partially preserved across these languages.

ADReSS-M provided a platform for contributions to the application of signal processing and machine learning methods for two tasks: multilingual Alzheimer’s dementia detection and cognitive score test prediction. The challenge also stimulated the discussion of machine learning architectures, novel signal processing features, feature selection and extraction methods, and other topics of interest to the growing community of researchers engaged in investigating the connections between speech and dementia. A total of 24 research teams from 14 different countries (Belgium, Canada, China, Denmark, India, Finland, Germany, Greece, Poland, Spain, South Korea, Sweden, U.K. and USA) took part in the challenge, with the majority (17) creating models and submitting results for both tasks.

The approaches adopted by the various research groups that entered the challenge were quite diverse. Feature extraction approaches ranged from acoustic feature extraction using standard feature sets such as eGeMAPS [18], to transcript generation through automatic speech recognition followed by linguistic feature extraction through pre-trained multilingual word embedding models, to task-specific feature engineering (representing speech intelligibility and different pause features, for instance), and combinations of these approaches, sometimes followed by further dimensionality reduction methods. Machine learning approaches included transfer learning using deep learning architectures, conventional machine learning algorithms such as support vector machines, logistic regression, random forests, gradient boosting, and late fusion methods involving combinations of these approaches. Feature fusion combining acoustic, paralinguistic and linguistic features was also often employed.

In what follows, we describe the ADReSS-M challenge’s modelling tasks, along with their evaluation metrics and ranking procedure, present the data sets in detail, describe our baseline models for the task, present the challenge’s results, including a ranking table with the five top-scoring submissions along with brief descriptions of the methods and approaches used by each of these submissions, present a summary of their contributions, and discuss future prospects for this area.

## II. RELATED WORK

Early research on language as an indicator of cognitive decline tended to favour the analysis of characteristics such as information content, comprehension of complexity, and semantic fluency as predictors of disease progression [19]. However, content-free features have also been explored in early research, such as by Roark et al. [20], who used natural language processing (NLP) and automatic speech recognition (ASR) to generate basic paralinguistic features (pause frequency and duration), and analysed audio recordings of 74 neuropsychological assessments to classify participants into groups of people with mild cognitive impairment (MCI) or normal cognition. Their best classifier obtained an area under the receiver operating curve (AUC) of 86% by including a combination of automated speech and language features and cognitive tests scores. Spontaneous speech has also been investigated, as in a study that used semi-structured interviews from 9 healthy participants, 9 with AD, 9 with frontotemporal dementia, 13 with semantic dementia, and 8 with progressive non-fluent aphasia, extracting 41 features including speech rate, and the mean and standard deviation of the duration of pauses, vowels, and consonants to build a classification model that achieved 88% accuracy [21]. In a more recent study [22] graph-based features encoding turn-taking patterns and speech rate [23] were extracted from the Carolina Conversations Collection [24] of spontaneous interviews of AD patients and healthy controls. This study obtained 85% accuracy in distinguishing dialogues involving an AD speaker from controls.

Other studies have combined linguistic and paralinguistic features [25], [26], using signal processing and machine learning to detect subtle acoustic signs of neurodegeneration which may be imperceptible to human diagnosticians. While some studies found that filled pauses (sounds like “hmmm”, etc.) could not be reliably detected by human annotators, and that detection improved by using ASR-generated transcriptions [27], recent work has shown that filled pauses are good predictors of cognitive difficulties [10]. The use of virtual agents as a data collection strategy for AD detection has also been investigated [28], reaching accuracy as high as 83% on dialogue, eye-tracking and video data collected from 29 Japanese participants by a virtual character.

As regards data sets, one of the most widely used resources is the Pitt Corpus [29]. Its picture description task is one of the few available datasets that contain spontaneous speech and clinical information. This dataset has been used in several studies [26], [30], [31]. These studies used different combinations of linguistic and acoustic features, ranging from simple descriptive statistics to more complex feature embedding representations for AD and MCI classification.

Although research on speech as an indicator of cognitive function has increased in recent years, it remains difficult to compare the different studies, even when restricted to the same data sources. The ADReSS challenges [8], [9], [13] were created to mitigate this problem. In these shared tasks, participants used the same datasets, which were balanced for age and sex and acoustically normalised. The various approaches proposed to tackle the ADReSS challenges included state-of-the-art deep learning and word embedding methods, and focused mainly on linguistic features extracted from the manually generated transcripts. The ADReSS [8] winning team, for instance, leveraged audio recordings to obtain information about pauses in speech, encoding them as punctuation [32] into ensembles built from features extracted from pre-trained language models (BERT [33] and ERNIE [34]), and obtained 89.58% accuracy.

There are currently very few papers that report investigations involving modelling of AD or MCI across different languages, and to our knowledge no multilingual benchmark data set or shared task in this area existed before ADReSS-M. Previous research compared the use of monolingual and multilingual pre-trained language models, and found that multilingual models exhibited better performance across English-Swedish data sets [35], and in English-Italian data sets [36]. Similarly, Guo et al. [37] employed cross-lingual data augmentation based on pre-trained transformer models to detect AD in English and Mandarin speakers, finding that a contrastive learning, cross-lingual augmentation approach outperformed monolingual augmentation. A study by Lindsay et al. [38] investigated multilingual modelling of AD in an English-French corpus, attempting to systematically select the most generalisable features. They found that features derived from semantic processing were the most generalisable features, while paralinguistic features had low generalisation potential. Also regarding the use of language-independent acoustic features, a recent study compared mono- and cross-lingual features for MCI detection in English and Hungarian, and found no significant difference in performance [39].

## III. THE ADRESS-M TASKS

The ADReSS-M challenge consisted of two prediction tasks to be attempted by the participants, namely:

1. a classification task (AD detection), where the models aimed to distinguish speech of participants with normal cognition (NC, or control condition) from speech of participants with AD or mild cognitive impairment (MCI), and
2. a cognitive test score prediction (regression) task, where participants were asked to create models for inferring the speaker’s Mini-Mental State Examination (MMSE) score based on speech data.

AD and MCI classes were determined according to clinical diagnosis criteria. In the case of probable AD diagnoses, some were substantiated by neuropathologic examination and others were confirmed by autopsy, as described by Becker et al. [29]. The MMSE is a short, psychometrically sound screening tool for measuring cognitive functioning (e.g., orientation, attention, memory, language, visuospatial abilities) with a maximum score of 30 points [40].

Both tasks involved processing the raw spontaneous speech signal, extraction of features, using whatever pre-processing methods the participant wished to use, and creating the predictive models. No speech segmentation or transcription were provided.

Participants could choose to do one or both tasks. They were provided with a training set and, two weeks prior to the paper submission deadline, were given access to test sets on which they could test their models. Up to five sets of results were allowed for scoring for each task per participant. All attempts had to be submitted together.

As the broader scientific goal of ADReSS-M was to gain insight into the nature of the relationship between speech and cognitive function across different languages, we encouraged participants to upload papers describing their approaches and results to a pre-print repository such as arXiv or medRxiv regardless of their ranking in the challenge, and asked them to share their code through a publicly accessible repository, if possible using a literate programming environment.

## IV. THE DATA SETS

**FIGURE 1.**
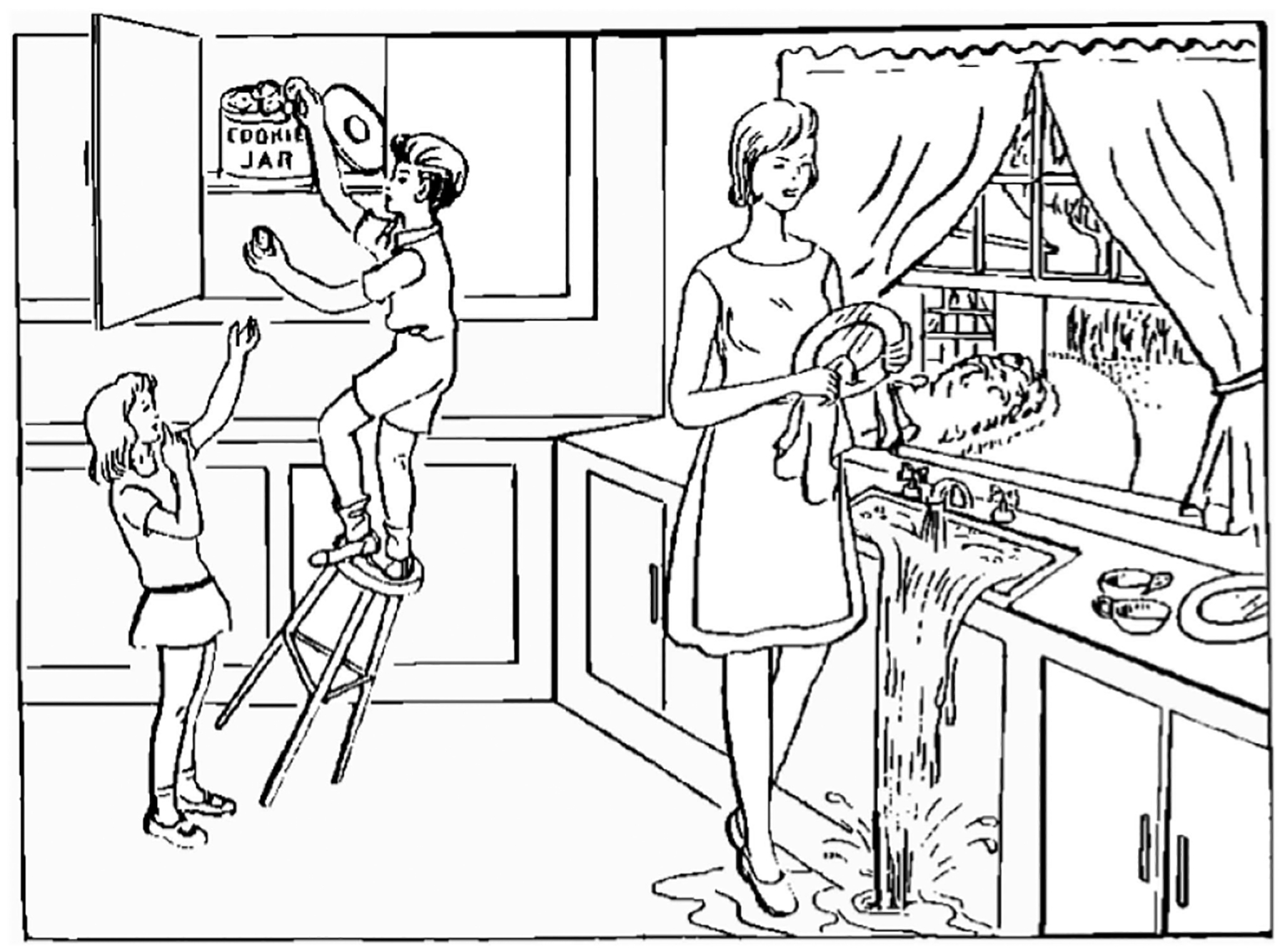
Cookie Theft picture from the Boston Diagnostic Aphasia Examination test, used to elicit connected speech for the English language data set.

The ADReSS-M data sets can be downloaded from DementiaBank at https://dementia.talkbank.org/ADReSS-M/, upon agreement with the terms and conditions of data sharing stipulated by that repository. The training data set consists of spontaneous speech samples corresponding to audio recordings of picture descriptions produced by cognitively normal subjects and patients with a (probable) AD diagnosis, who were asked to describe the Cookie Theft picture from the Boston Diagnostic Aphasia Examination test [29]. The participants were all native speakers of English, and were asked to describe the picture shown in Fig. 1.

The test set consists of spontaneous (connected) speech descriptions of a different picture, in Greek. The recordings therefore were in one of these languages, and contained speech produced by native speakers. Participants were initially allowed access only to the training data (in English) and some sample Greek data (8 recordings) for development purposes.

**FIGURE 2.**
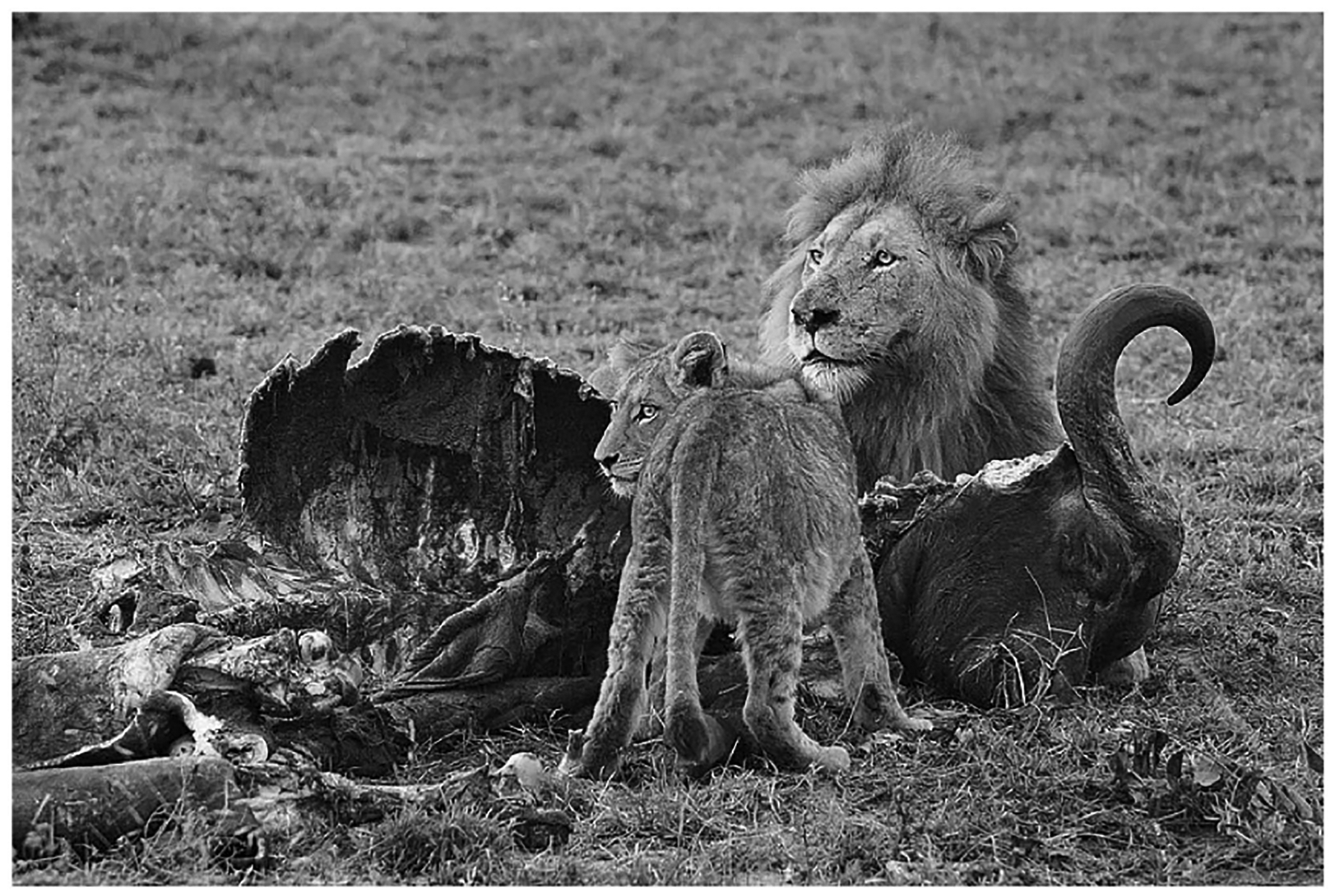
Image used in the Greek language picture description task (photograph by Luca Galuzzi, converted to grayscale by S Luz with an average HSI intensity saturation filter; licensed under CC BY-SA 2.5, https://creativecommons.org/licenses/by-sa/2.5/deed.en).

The Greek recordings assess participants’ verbal fluency and mood using a picture which the participant describes while looking at it. The assessor first shows the participant a picture representing a lion lying with a cub in the desert while eating, as shown in Fig. 2. The assessor then asks the participants to give a verbal description of the picture in a few sentences. The original purpose of this task was to evaluate the participant’s ability to generate coherent and descriptive language while also gaining insights into their mood as well as cognitive and emotional responses. By analysing the language used to describe the picture, researchers can assess the participant’s verbal fluency, vocabulary, syntax, and overall linguistic capabilities. Additionally, the context in which the data were collected is crucial to understanding the significance of the task and its findings. This particular task was conducted as part of a psychological and linguistic research study carried out to examine language processing, cognitive abilities, emotional responses and mood-related factors, and to explore potential connections between language and cognitive states through this assessment.

**FIGURE 3.**
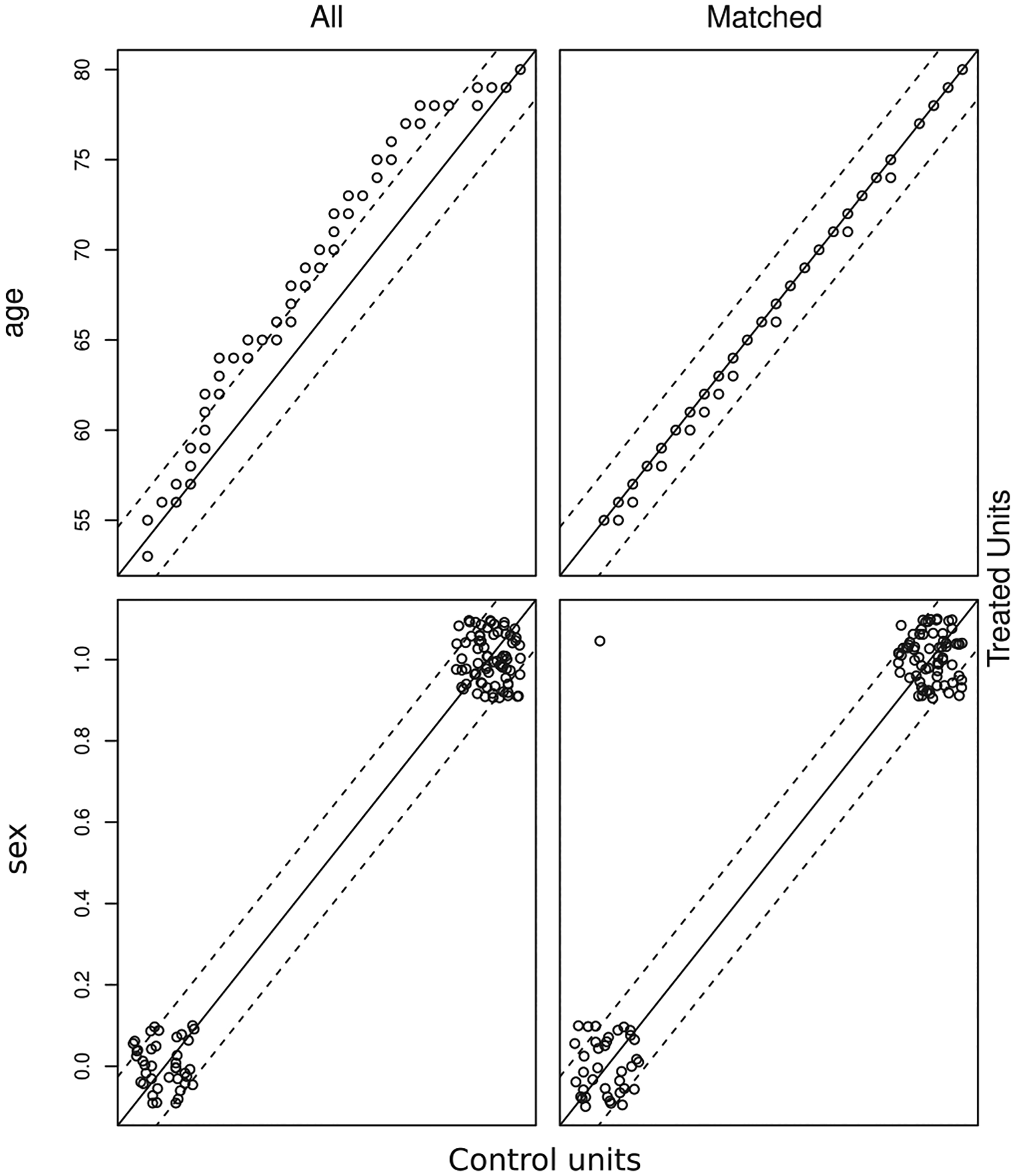
eQQ plots for the original data set and corresponding balanced training data set.

The training data set was balanced with respect to age and sex so as to eliminate potential confounding and bias. As we employed a propensity score matching approach [41] we did not need to adjust for education as this variable correlates with age and sex, which suffice as an admissible adjustment (see [42, pp 348–352]). Note, however, that the education variable could still be used for predictive modelling. The data set was checked for matching according to scores defined in terms of the probability of an instance being treated as AD given covariates age and sex estimated through logistic regression, and matching instances were selected. All standardised mean differences for the covariates were below 0.1 and all standardised mean differences for squares and two-way interactions between covariates were below 0.15, indicating adequate balance for those covariates. The empirical quantile-quantile (eQQ) plots for the original and balanced data sets [43] are shown in Fig. 3. The matched data eQQ plots show instances near the diagonal and clear separation of the nominal variables, which indicate good balance. The top left plot shows that the age distribution in the full (non-matched) source data set had an age distribution skewed towards older ages for the MCI/AD, showing some level of balance only at the extremes (youngest and the oldest of the old participants). The top right plot shows that the matching procedure produced a well balanced set across all quantiles. The bottom plots show the distributions of the sex variable. As this is a binary variable, the data points are concentrated at the extremities of the main diagonal, with any unmatched data appearing as off diagonal dots (at the other corners of the plot). As can be seen on the bottom-left plot, the sex variable was already well balanced in the source dataset, and the bottom-right plot shows that balance was preserved by the matching process.

The mean age, MMSE, and the numbers of NC to AD participants in the respective categories are shown in Table 1. The overall ratio of AD to NC for the training data is 22:23.

The test set had similar statistical characteristics, but slightly higher average ages and MMSE scores for each category. The detailed composition of the test set is shown in Table 2. The AD to NC ratio for the test set was 22:24.

The training set audio recordings were distributed in MPEG audio layer 2/3 format, with a sample rate of 44,100 Hz and bit rate of 128 kb/s. The test set audio was encoded in 16-bit Signed Integer PCM format, with a sample rate of 22,050 Hz.

## V. EVALUATION METRICS

The classification task is evaluated in terms of accuracy (*A*), specificity (*Sp*), sensitivity (*ρ*) and *F*_1_ scores. These metrics were computed according to (1)–(5).

(1) $$A=\frac{T_{n}+T_{p}}{N}$$

(2) $$Sp=\frac{T_{n}}{T_{n}+F_{p}}$$

(3) $$F_{1}=2\frac{\pi \times \rho }{\pi +\rho }$$

where *N* is the number of patients, *T*_*p*_ is the number of true positives, *T*_*n*_ is the number of true negatives, *F*_*p*_ is the number of false positives, *F*_*n*_ is the number of false negatives. The *F*_1_ scores is the harmonic mean of sensitivity and positive predictive value, or precision (noted *π*), computed as shown in (4) and (5).


(4) $$\rho =\frac{T_{p}}{T_{p}+F_{n}}$$

(5) $$\pi =\frac{T_{p}}{T_{p}+F_{p}}$$

For the regression task (MMSE prediction), the metrics used are the coefficient of determination and root mean squared error (RMSE), as set out in (6) and (7), respectively, where where ŷ_*i*_ is the predicted MMSE score, *y*_*i*_ is the patient’s actual MMSE score, and *ȳ* is the mean score.


(6) $$R^{2}=1-\frac{\sum_{i=1}^{N}{\left({\hat{y}}_{i}-y_{i}\right)}^{2}}{\sum_{i=1}^{N}{\left({\hat{y}}_{i}-\bar{y}\right)}^{2}}$$

(7) $$\text{RMSE}=\sqrt{\frac{\sum_{i=1}^{N}{\left({\hat{y}}_{i}-y_{i}\right)}^{2}}{N}}$$

The ranking of submissions was based on accuracy scores for the classification task (task 1), and on RMSE scores for the MMSE score regression task (task 2). The top 5 models comprised:

1. The two top performing (most accurate) teams for the classification task.
2. The two top performing (least RMSE) teams for the MMSE regression task.
3. The team that performed best on average for the two tasks, chosen according to the formula set out in (8), where *T*_*i*_ is the total score of team *i* and *T* is the total number of teams in the challenge. If a team chose not to submit results for a task, its score for that task was set to 0.
(8) $$T_{i}=\frac{A_{i}}{\sum_{j}^{T}A_{j}}+1-\frac{{\text{RMSE}}_{i}}{\sum_{j}^{T}{\text{RMSE}}_{j}}$$

Ties were broken by averaging performance over all attempts. These criteria were applied so that the rank resulted in 5 different teams. Thus, if one team was selected as a top team under one of the criteria, it would not be selected as a top team in another. In such cases, the next top-performing team would be selected. This was done in order to avoid a situation in which the top-5 teams overall happened to have done well at one task but had mediocre performance at the other, while a team lower on the overall rank had superior performance at the latter task.

## VI. BASELINE MODELS

We created baseline models for each task to give the participants an idea of what the use of standard signal processing and machine learning methods could achieve for these tasks on the provided data sets.

In creating these models, we first normalised the volume of the audio files using FFMPEG’s [44] implementation EBU R128 scanner filter [45]. A sliding window of 1 s, with no overlap, was then applied to the audio recordings, and eGeMAPS features were extracted over these frames. The eGeMAPS feature set [18] is a basic set of acoustic features designed to detect physiological changes in voice production. The minimalistic acoustic parameter set consists of eighteen low-level descriptors (LLD) arranged according to parameter groups: pitch, jitter, formant frequency, shimmer, loudness, harmonics-to-noise ratio, spectral (balance) parameters, harmonic difference, and energy/amplitude related parameters. A symmetric moving average filter is used to smooth these LLDs across time. The arithmetic mean and coefficient of variation are then taken for these 18 LLDs, resulting in 36 parameters. Pitch and loudness are given additional functionals (i.e. percentile and rising and falling slopes) yielding a total of 56 parameters. The extended set includes seven further LLDs, fourteen additional descriptors, the arithmetic mean of spectral flux in unvoiced areas, the arithmetic mean of spectral flux and MFCC 1–4 in voiced parts, and the equivalent sound level, resulting in the 88 eGeMAPS features, in total.

**FIGURE 4.**
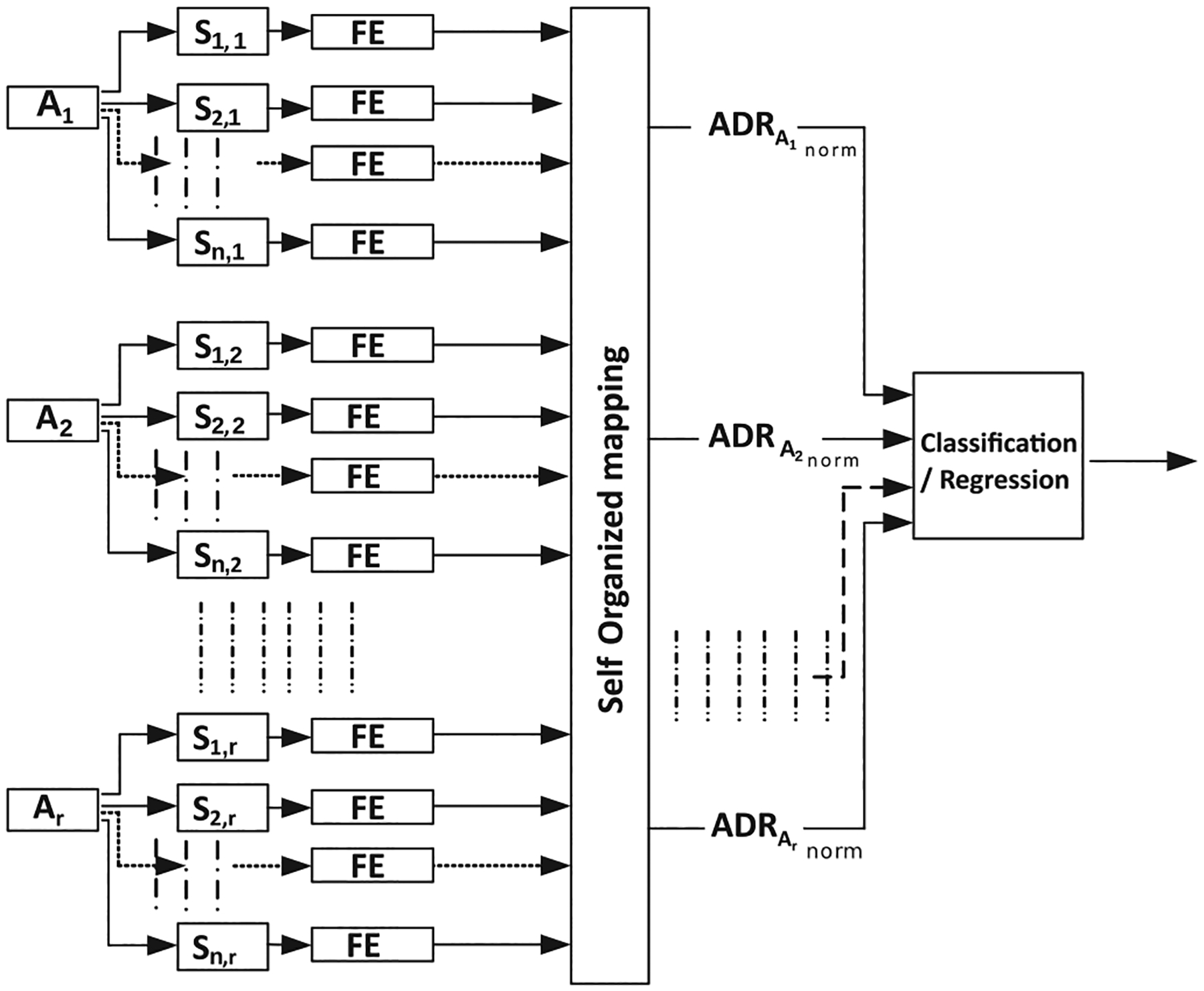
ADReSS-M baseline system architecture.

Given the eGeMAPS features, we applied the active data representation method (ADR) [26] to generate a frame level acoustic representation for each audio recording. The ADR method has been used previously to generate large scale time-series data representation. It employs self-organising mapping (SOM) to cluster the original acoustic features into dimensions that represent the number of clusters (“neurons”) in the map produced by SOM. It then computes histogram representation of these clusters (as shown in (9) and (10)) for each audio file (i.e. *A*_*i*_) and their first-order derivative features (mean and standard deviation features [26], where the rate of change is given by an approximation of the derivative ((9), which are then normalised ((10)) for use in the ADR model (Fig. 4).


(9) $$vADR_{Ai}=\frac{\partial cADR_{Ai}}{\partial t}$$

(10) $$nADR_{Ai_{\text{norm}}}=\frac{nADR_{Ai}}{{\left\|nADR_{Ai}\right\|}_{1}}$$

This method is entirely automatic in that no speech segmentation or diarisation information is provided to the algorithm.

For the AD detection task (task 1), we employed a Naïve Bayes classifier with kernel smoothing estimation. The ADR for feature extraction was optimised using grid search (*C* = 5, 10, **15**, 20, 25, where *C* stands for the number of SOM clusters, as described above). In previous work, we used 2(*C* + 2) features, which corresponded to two ADR sets (nADR and dADR, the second of which characterised frame duration), each ADR consisting of *C* features and its respective mean and standard deviation, plus age and sex [26]. However, in the present study, as the duration is the same for all frames, we used only *C* + 2 features (nADR, mean and standard deviation) plus age and sex. Thus the ratio of features to training audio samples was 19:237. With this data representation we achieved accuracy of 75.00% and **73.91%** on sample and validation data respectively. On the test set, specificity was 79.2%, precision was 75%, sensitivity was 68.2%, and *F*_1_ was 71.4%.

For the MMSE regression task (task 2), we employed a support vector machine (SVM) regressor model with an RBF kernel with box constraint set to one, using a sequential minimal optimisation solver. The ADR procedure for feature extraction was optimised using grid search (*C* = 5, 10, 15, 20, **25**). This model achieved an RMSE of 3.887 (*r* = 0.348) and **4.955** (*r* = 0.273) on sample and test data respectively using 25+2 ADR, age and sex features per recording. The ratio of features to training audio samples was also 29:237.

The source code for the data set generation procedure and for the baseline system is available at https://gitlab.com/luzs/madress-2023, with access granted upon request.

## VII. RANK OF SUBMISSIONS

The submissions were ranked according to the procedure described in Section V. The scores for the top-5 teams (excluding the baseline system) are shown in Table 3.

The top scoring team, from the Dept of Computer Engineering at Konkuk University and VOINOSIS Inc, South Korea, employed a novel complementary and simultaneous ensemble algorithm (CONSEN) on acoustic and disfluency features, exploring correlations between AD and MMSE predictions to improve performance [46]. Disfluency, pause and speech rhythm features have long been used to assess human performance [47], and have been recently applied to AD detection to good effect [10], [48]. The team that came in second place employed a mixed-batch transfer learning approach for both tasks, applied to eGeMAPS acoustic features [49]. The third highest scoring team explored a wider number of acoustic feature extraction methods, employing an XGBoost classifier for the classification task and SVM and XGBoost regressors for MMSE prediction [50]. The fourth ranked team employed an automatic speech recognition system to derive speech intelligibility features based on confidence scores assigned by the system, which along with word-level duration and pause features formed the input for logistic regression and SVM regression models for tasks 1 and 2, respectively [51]. The team the came in fifth place fused linguistic and acoustic features extracted through speech recognition and pre-trained word embedding and acoustic embedding models and employed neural networks consisting of two fully connected layers and SVMs for classification and regression [52].

**FIGURE 5.**
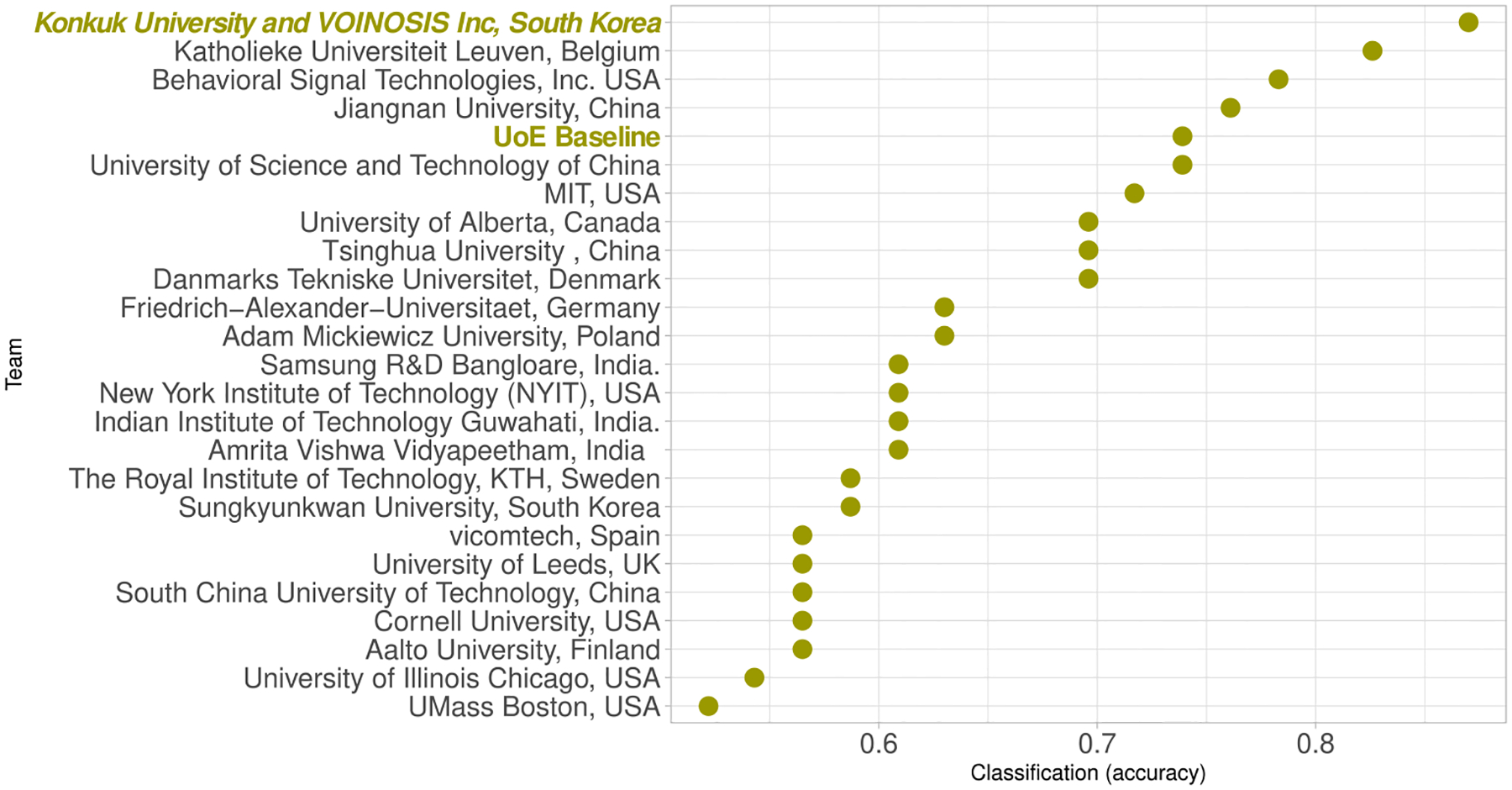
AD detection accuracy results.

The overall accuracy ranking for the participants is shown in Fig. 5. It can be observed, in this dot chart, that there is a considerable gap between the two top-scoring teams and the remaining teams. This reflects their effective use of transfer learning techniques, as well as the ability to identify language-independent features.

**FIGURE 6.**
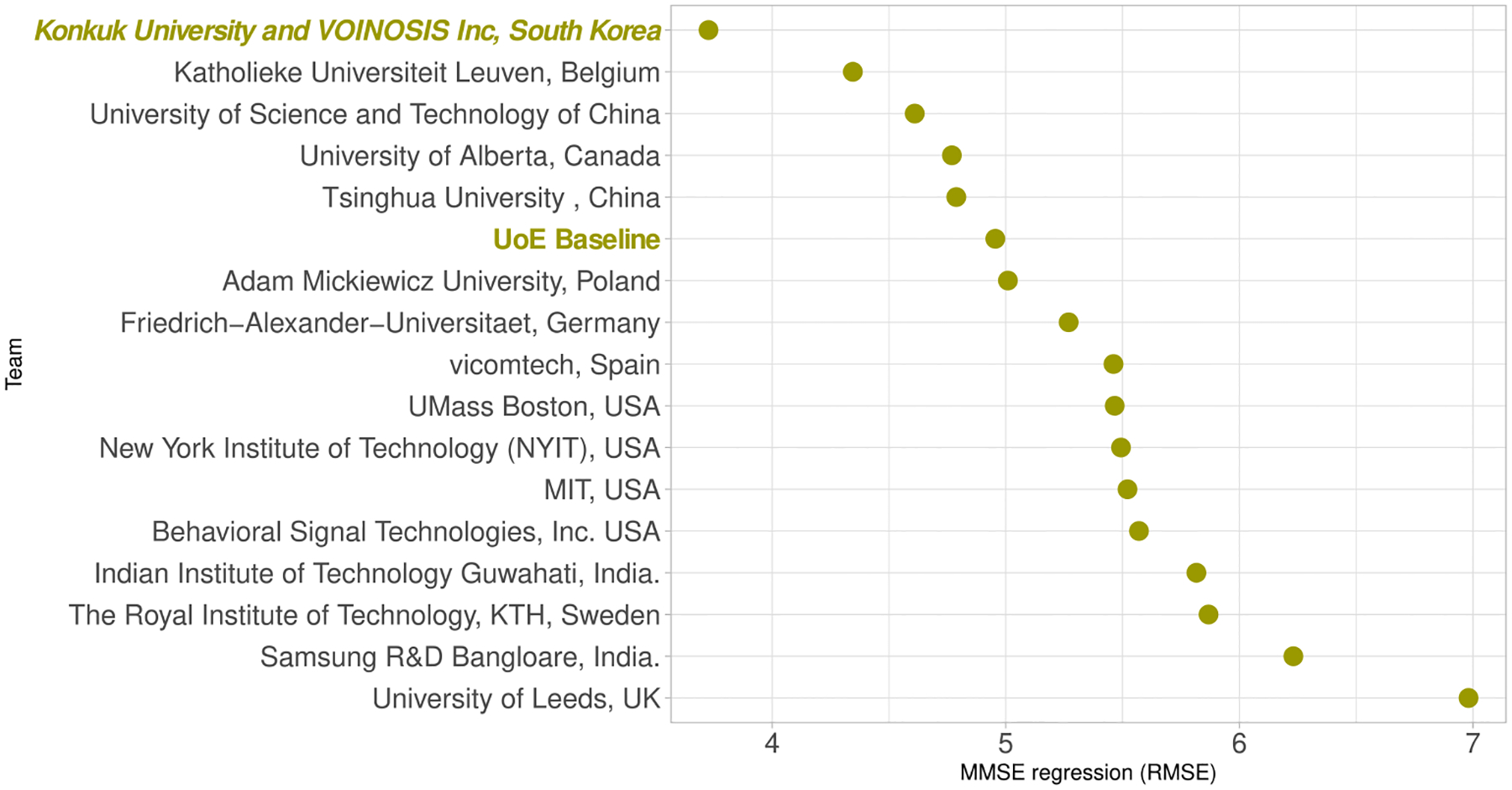
MMSE regression results.

A similar pattern can be discerned in the chart depicting the regression results (Fig. 6) where the gap between the top scoring team and the remaining teams is even more pronounced. This underscores the effectiveness of the approach of using learning of MMSE scores to leverage classification learning, employed by the winning team.

## VIII. DESCRIPTIONS OF THE TOP-5 SUBMISSIONS

Jin et al. [46] conducted a series of experiments using acoustic, disfluency and fusion of acoustic and disfluency features. They showed that the disfluency feature provides better results than acoustic features and generalises well across languages. They proposed an ensemble algorithm (CONSEN) which achieved the best-performing results using the fusion of disfluency and acoustic features with an accuracy of 87.0% in AD detection and 3.727 RMSE in MMSE prediction. The unique feature of this top-scoring approach was its leveraging of MMSE prediction as a means to improve AD detection accuracy. While this approach would not be feasible were training data for cognitive testing not available, it suggests an interesting way of combining speech-based cognitive assessment with better established tests of cognitive function currently in clinical use.

Tamm et al. [49] created models using a sequence of acoustic features and covariates (age, sex, and education). The models were first trained on English data, and then transferred to Greek using mixed-language batches and parameter averaging. Results yielded 82% accuracy for AD detection and an RMSE of 4.345 for MMSE score prediction on the test set. For the classification task, the best model had 91.7% specificity, 88.9% precision, 72.7% sensitivity and an F1-score of 80.0%. The distinguishing characteristic of Tamm et al.’s approach is their use of the same deep learning architecture for both tasks. Their network architecture consisted of batch normalisation of input features, attention weights computed by two feed-forward layers with dropout and ReLU activation.

Mei et al. [50] provide insights into the methodologies, techniques, and algorithms employed by the USTC team to tackle the ADReSS-M Challenge. They discuss their system’s architecture, data preprocessing, feature extraction methods, and machine learning or deep learning models used for emotion recognition in speech. The unique characteristics of the approach described are the use of a 10-dimensional feature set for distinguishing among pauses, following the method proposed in a previous AD detection challenge [48], the fusion of several low-level paralinguistic descriptors used for extraction and fine-tuning of a pre-trained wav2vec2 model [53]. The XGBoost classifier [54] achieved 73.9% accuracy, and the pre-trained bilingual model achieved up to 87.5% in validation against the Greek language samples provided for training. The results indicate that using balanced, low-pass filtered, bilingual speech data in fine-tuning pre-trained models and classifier training could be beneficial to multilingual AD detection.

Shah et al. [51] investigated language-agnostic speech representations, which are speech features or characteristics that can be effectively applied across different languages, without requiring language-specific adaptations [55]. The researchers focused on using domain knowledge, likely related to the specific characteristics of AD, to develop and evaluate these speech representations for the purpose of detecting the early cognitive changes across the AD spectrum. The study explored various machine learning techniques to learn meaningful representations from speech data, considering language-agnostic aspects to ensure the model’s generalisation across multiple languages. The findings of this research could contribute to the development of robust and language-independent diagnostic tools for AD, making it easier to identify potential patients regardless of their native language. The paper presents a concise overview of the researchers’ methodology, experimental results, and implications for future research directions in the domain of speech-based AD detection.

Chen et al. [52] made use of three processing streams in their approach to the ADReSS-M tasks. For the extraction of paralinguistic features, they used three different feature sets extracted through the openSMILE toolkit [56] and pre-trained models. They applied SVM to each separately to perform classification and prediction. The best *F*_1_ score for these three analyses was 0.72 for the IS10-Paralinguistics feature set [57]. For an analysis based on pre-trained acoustic features, they used the XLSR-53 model [58]. Although that model has been trained on 53 languages, it does not include Greek and this could have led to a weaker performance for this method. Using the Whisper speech recognition model, they produced English texts from the Greek audio which they used to train a RoBERTa model. This method produced a lower *F*_1_ score of 0.55 due to inconsistencies between the pictures described in Greek and those for English. Features from both the XLSR-53 model and the RoBERTa model used a two level fully connected network to generate values for classification and regression.

## IX. DISCUSSION

ADReSS-M attracted the participation of a large number of teams from leading research labs from across the world, evidencing the relevance of the emerging field of research on speech-based digital biomarkers for AD in general, and on methods that generalise across languages in particular. The diversity of approaches presented by the participating teams, including proposals for novel acoustic feature sets, the use of pre-trained models, the combination of automatic speech recognition and multilingual embedding models, the use of transfer learning, and a novel ensemble learning method that combines the diagnosis and the cognitive score prediction learning tasks will hopefully open new avenues for further explorations in this area.

Despite the fact that ADReSS-M focused on a multilingual or cross-lingual learning setting, the submissions to the challenge tended to follow the trends set in previous shared tasks aimed at assessing cognitive function through analysis of speech [8], [9], [12] as regards feature engineering and feature extraction. Considering the small size of the ADReSS-M data set and the fact that the picture descriptions were different in the training and test sets (not only in language but also in content, as the pictures were different), we expected that the proposed models would rely on more abstract acoustic features rather than on lexical or structural linguistic features, as the former are presumably less language-dependent than the latter [26], [59], [60]. Indeed this was the case for most submissions, as four of the top-scoring teams [46], [49], [50], [51] employed acoustic features exclusively (even though in some cases ASR output was employed to derive dysfluency and pause features). However, some of the submitted models, including one of the top-5 [52] employed linguistic features, either by themselves or in combination with acoustic and paralinguistic features.

Among the proposed acoustic models, the majority employed pre-trained models such as wav2vec2 [61] and Whisper [62] as a means of extracting acoustic features. Such approaches have been employed successfully in AD detection tasks, from the first ADReSS challenge, where transformer-based language models were widely used in combination with paralinguistic information [10], [63], [64] to recent work presented at ICASSP 2023 [65] which compared several large-scale, pre-trained acoustic and language models for the original (monolingual) ADReSS classification task. Acoustic features derived through feature engineering, notably some commonly used openSMILE-generated feature sets were also used, and achieved good results [49], in combination with demographic information. It is noteworthy that the use of features that characterise speech dysfluency proved effective in several models, confirming the findings of models trained and tested on monolingual data (e.g. [10], [66]) in previous challenges. Therefore it seems fair to conclude that these features are both effective and generalisable across languages.

As regards the classification and regression algorithms employed by the participating teams, both conventional machine learning algorithms — such as classifier ensembles (including Random Forests), gradient boosting (including XGBoost), SVM, SVR, and logistic regression — and deep neural networks. In some cases [51], [65], these methods were used for feature selection in addition to classification and regression.

While we believe ADReSS-M provides a useful standard benchmark for assessment of cognition across the two languages in our data set, we acknowledge that it also has limitations. As with all shared machine learning tasks, focusing the attention of a large community on a single task and data set poses the risks associated with “over testing” at the community level, namely, that results might be due to particular choices of parameters rather than to general characteristics of language and their relation with cognition. More research is needed on the mechanisms underlying cognitive decline in Alzheimer’s disease and how these mechanisms might translate to linguistic and phonological behaviour. This is a complex undertaking, which we hope ADReSS-M and similar task might contribute to facilitating. Within the task itself, comparability of results is somewhat problematic due to the fact that many different approaches were employed, some of which leveraged information that was available for both tasks (classification and regression) rather than the individual task in question. Prediction of MMSE scores can obviously help prediction of AD, and the fact that MMSE scores were available benefited those teams that chose to exploit them. While the challenge’s rules did not preclude the use of such strategies, and in fact their use illustrates interesting possibilities for ensemble learning which we had not foreseen, MMSE information may not always be available in practical situations. Finally, we believe the ICASSP regulations regarding accepting only papers from the five top-scoring teams risked excluding interesting approaches which, while not scoring well in the tasks, might have provided interesting insights into the problem of cognitive assessment across languages. This is an issue future challenge organising committees might wish to consider.

## X. CONCLUSION

Computational analysis of spontaneous connected speech has the potential to enable novel applications for speech technology in longitudinal, unobtrusive monitoring of cognitive health. By focusing on AD recognition using spontaneous speech, the ADReSS-M signal processing grand challenge provided a platform for the investigation of alternative to neuropsychological and clinical evaluation approaches to AD detection and cognitive assessment. Furthermore, we expect that the multilingual resources and models provided by ADReSS-M will allow the investigation of features that might generalise across languages, extending the applicability of these models in future. In keeping with the objectives of AD prediction evaluation, the ADReSS-M challenge provided a statistically balanced data set to mitigate common biases often overlooked in evaluations of AD detection methods, including repeated occurrences of speech from the same participant, variations in audio quality, and imbalances of sex, age and educational level. We hope this might serve as a benchmark for future research on multilingual AD assessment.

## Figures and Tables

**TABLE 1.** Descriptive Statistics for the ADReSS-M Training Set (English) by Diagnostic Category (Dx) and Sex

Dx	Sex	n	Age (sd)	MMSE (sd)
NC	Female	75	65.6 (6.22)	29.0 (1.29)
NC	Male	40	67.7 (7.12)	28.9 (0.91)
AD	Female	70	69.9 (6.40)	17.4 (5.10)
AD	Male	40	68.4 (7.68)	18.7 (6.08)

Abbreviations: N = Number of Participants, Sd = Standard Deviation, MMSE = Mini-Mental State Examination.

**TABLE 2.** Descriptive Statistics for the ADReSS-M Test Set (Greek) by Diagnostic Category (Dx) and Sex

Dx	Sex	n	Age (sd)	MMSE (sd)
NC	Female	18	66.5 (6.66)	29.0 (1.03)
NC	Male	6	63.5 (9.38)	28.7 (1.63)
AD	Female	17	72.5 (6.97)	20.5 (4.61)
AD	Male	5	72.4 (8.08)	20.8 (4.66)

**TABLE 3.** Ranking of Teams Results by Overall Composite (T) Scores (Combined Classification and Regression Results)

Rank	Team	Overall (T)	Detection (A)	MMSE (RMSE)
1	Dept of Computer Engineering at Konkuk University and VOINOSIS Inc, South Korea	1.011	0.870	3.727
2	Katholieke Universiteit Leuven, Belgium	1.002	0.826	4.345
3	University of Science and Technology of China	0.994	0.739	4.610
–	University of Edinburgh Baseline	0.990	0.739	4.955
4	University of Alberta, Canada; ILSP, Athena Research Centre, Greece	0.989	0.696	4.769
5	Tsinghua University, China	0.989	0.696	4.788
